# SARS-CoV-2 Infection Incidence and Outcome Before and After Full Vaccination in Patients With Monoclonal Gammopathy of Undetermined Significance

**DOI:** 10.1097/HS9.0000000000000800

**Published:** 2022-11-09

**Authors:** Nicola Sgherza, Daniela Di Gennaro, Paola Curci, Rita Rizzi, Daniela Roccotelli, Maria Croce, Martina Avantaggiato, Loredana Ruga, Vanda Strafella, Angelantonio Vitucci, Antonio Palma, Antonella V. Russo Rossi, Teresa Troiano, Angela M. V. Larocca, Maria Chironna, Silvio Tafuri, Francesco Albano, Pellegrino Musto

**Affiliations:** 1 Hematology and Bone Marrow Transplantation Unit, AOUC Policlinico, Bari, Italy; 2 Department of Emergency and Organ Transplantation, “Aldo Moro” University School of Medicine, Bari, Italy; 3 Department of Clinical Patology, AOUC Policlinico, Bari, Italy; 4 Hygiene Unit, AOUC Policlinico, Bari, Italy; 5 Department of Biomedical Sciences and Human Oncology, “Aldo Moro” University School of Medicine, Bari, Italy

Epidemiological studies have previously reported that patients with monoclonal gammopathy of undetermined significance (MGUS) may have an increased risk of developing viral infections.^1,2^ Regarding specific antiviral immunological response, no significant differences between MGUS and normal controls have been detected about herpesviruses HSV1, cytomegalovirus (CMV), and Epstein-Barr virus, while the median titer of antivaricella-zoster virus IgG was found significantly lower in MGUS patients.^3^ It has been also speculated that the intrinsic immune dysregulation exhibited by MGUS subjects may contribute to determine a suboptimal serological response to vaccines, including those anti-SARS-CoV-2.^4^ In this setting, the capacity to produce neutralizing antibodies after anti-SARS-CoV-2 vaccine (2 doses of BNT162b2 or 1 dose of AZD1222) in patients with different types of plasma-cell dyscrasia was reported to be not significantly different between MGUS subjects and healthy controls (HCs).^5^ These data were further confirmed by another study,^6^ carried out on fully vaccinated patients (2 doses of BNT162b2 or mRNA-1273 or 1 dose of Ad26.COV2.S) with asymptomatic precursor stages of multiple myeloma. Contrarily to smoldering myeloma, an attenuated antibody response in MGUS patients was not observed. The role of at least two doses and that of a “booster” administration of anti-SARS-CoV-2 vaccines has been further underlined in multiple myeloma but only marginally in MGUS.^7,8^

We and others recently showed that patients with MGUS had the same risk of SARS-CoV-2 infection and a similar clinical outcome compared to age- and sex-matched HCs during the first wave of the COVID-19 pandemic, before the availability of anti-SARS-CoV-2 vaccines.^9,10^ Here, we report that fully vaccinated individuals with MGUS maintain an analogous incidence of SARS-CoV-2 infection, but also show a significant improvement in clinical outcomes of COVID-19 compared to not vaccinated patients, formally proving, for the first time, the efficacy of anti-SARS-CoV-2 vaccines in this population of patients.

We obtained retrospective information from 86 individuals found to be SARS-CoV-2-positive until March 2022, among 1060 vaccinated MGUS patients analyzed after at least a second dose of anti-SARS-CoV-2 vaccine received between March and December, 2021, with a median follow-up of 300 days (range 30–454). Patients with monoclonal gammopathies of clinical significance, previous SARS-CoV-2 infection, only one dose of vaccine received or SARS-CoV-2 infection after one dose, as well as not vaccinated (no-vax) subjects, were excluded from the final analysis. SARS-CoV-2 infection was confirmed by rapid antigen test or RT-PCR on nasopharyngeal swabs.

Clinical data were collected from a review of medical records and regarded, in particular, age, comorbidities (cardiovascular, pulmonary or renal diseases, diabetes, and nonhematological cancers), the presence of symptoms (in detail: fever or chills, cough, shortness of breath or difficulty breathing, fatigue, muscle or body aches, headache, loss of taste or smell, sore throat, congestion or runny nose, nausea or vomiting, diarrhea), hospitalization, hospitalization in intensive care unit (ICU), and outcome (alive/dead). Additional information were extracted from “Infections Regional Information System (IRIS)-Puglia,” a regional platform where authorized medical health workers can view the results of the nasopharyngeal swabs for SARS-CoV-2 performed, along with other clinical information. Statistical analyses were carried out using GraphPad Prism version 8.3.0 (GraphPad Software Inc., San Diego, CA). The study was conducted within the context of the ClinicalTrials.gov Identifier NCT04352556.

Clinical characteristics of not vaccinated MGUS have been previously reported in detail^9^ and are summarized and compared with those of vaccinated patients in Table 1. Of the 86 vaccinated SARS-CoV-2-positive patients, 53 (61.6%) were men, while the mean (SD) age was 65.9 (±13.4) years. One, 2, and ≥3 of evaluated comorbidities were reported in 32 (37.2%), 10 (11.6%), and 14 (16.3%) of patients, respectively The most frequent MGUS-isotype was IgG (74.4%), followed by IgM (16.3%), IgA (7%), and biclonal (2.3%). Immunoparesis was present in 10 patients (11.6%), absent in 68 (79.1%), not available in 8 (9.3%). Most of patients (96.5%) were at low or low-intermediate risk, according to the Mayo Clinic prognostic model.

**Table 1:** Characteristics of MGUS Patients With SARS-CoV-2 Infection and COVID-19 Outcome Before and After anti-SARS-CoV-2 Vaccination

	Prevaccination^a^	Postvaccination	*P* value
Total number of pts	91	86	–
Mean age, y ± SD (range)	65.6 ± 13.3 (29-89)	65.9 ± 13.4 (31-90)	0.84
Gender (male/female)	42/49	53/33	0.05
Comorbidities, n (%)^b^			
0	29 (31.9)	30 (34.9)	0.67
1	30 (32.9)	32 (37.2)	0.55
2	19 (20.9)	10 (11.6)	0.09
≥3	13 (14.3)	14 (16.3)	0.71
Mean number, n ± SD (range)	1.3 ± 1.3 (0–5)	1.035 ± 1.068 (0–3)	0.29
MGUS subtype, n (%)	(available in 86 patients)		
IgG	63 (73.2)	64 (74.4)	0.86
IgA	5 (6.0)	6 (7.0)	1.0
IgM	9 (10.4)	14 (16.3)	0.26
Biclonal	8 (9.3)	2 (2.3)	0.09
LC only	1 (1.1)	0	1.0
MGUS risk,^c^ n (%)	(available in 47 patients)		
0 Low	22 (46.8)	54 (62.8)	0.07
1 Low-intermediate	22 (46.8)	29 (33.7)	0.13
2 High-intermediate	3 (6.4)	3 (3.5)	0.44
Total SARS-CoV-2-positive MGUS, n (%)	91/1454 (6.2)	86/1060 (8.1)	0.07
COVID-19 outcome			
Presence of symptoms, n (%)	54 (59.3)	23 (26.7)	< 0.001
Hospitalization, n (%)	19 (20.9)	2 (2.3)	< 0.001
Hospitalization in ICU, n (%)	10 (11.0)	1 (1.2)	0.009
Death due to COVID-19, n (%)	8 (8.8)	1 (1.2)	0.03
Mean number of days from the last vaccine dose to SARS-CoV-2 infection, ± SD (range)	NA	103 ± 80.3 (2-285)	NA

a Data extracted by Sgherza et al.^9^

b Comorbidities evaluated included cardiovascular disease, pulmonary disease, diabetes, and nonhematological cancers.

c Rajkumar SV et al. Serum free light chain ratio is an independent risk factor for progression in monoclonal gammopathy of undetermined significance. *Blood* 2005;106(3):812–817.

ICU = intensive care unit; MGUS = monoclonal gammopathies of undetermined significance; NA = not applicable; pts = patients; SD = standard deviation.

Overall, the incidence of SARS-CoV-2 infection was not significantly different between not vaccinated and vaccinated MGUS patients (Table 1). A case of reinfection was found. The patient was a 65-year-old woman, with IgGk, low-intermediate risk MGUS, positive at first on January 2021 (not yet vaccinated), and then in January 2022, 25 days after the second dose of BNT162b2 vaccine. In both circumstances, infection was asymptomatic.

Rates of symptoms, hospitalization, hospitalization in ICU, and deaths were instead significantly lower in vaccinated than in not vaccinated MGUS patients (Table 1). In particular, only 2 hospitalizations, one of which in ICU, and one death were reported among vaccinated MGUS subjects. The patient hospitalized in ICU and discharged alive, was a 75 years-old man with IgA lambda-MGUS and hypertension. The dead patient was an 89 years-old man with IgM kappa-MGUS, asbestosis, and implantable cardioverter defibrillator; both these patients had received 3 doses of BNT162b2 vaccine. Notably, SARS-CoV-2 incidence and related symptoms were highly more frequent among patients after 2 doses than in those treated with 3 doses, while the mean number of days between the last dose of vaccine and infection was inferior (Table 2).

**Table 2:** Effects After 2 and 3 anti-SARS-CoV-2 Vaccine Doses in MGUS Patients

	Two Doses	Three Doses	*P* Value
Vaccine sequence in SARS-CoV-2-positive MGUS pts, n	BNT162b2 mRNA × 2, 31 ChAdOx1 nCoV-19 × 2, 9 mRNA-1273 × 2, 2	BNT162b2 mRNA × 3, 28 ChAdOx1 nCoV-19 × 2/mRNA-1273, 7 BNT162b2 mRNA × 2/mRNA-1273, 4 ChAdOx1 nCoV-19 × 2/BNT162b2 mRNA, 3 mRNA-1273 × 2/BNT162b2 mRNA, 2	NA
SARS-CoV-2 infection, n (%)	42/156 (26.9)	44/904 (4.9)	<0.001
COVID-19 outcome			
Presence of symptoms, n (%)	17/42 (40.5)	6/44 (13.6)	0.007
Hospitalization, n (%)	0	2 (4.5)	NA
Hospitalization in ICU, n (%)	0	1 (2.3)	NA
Death due to COVID-19, n (%)	0	1 (2.3)	NA
Mean number of days from last vaccine dose to SARS-CoV-2 infection, ±SD (range)	47.3 ± 41.9 (2–245)	171 ± 60.8 (2–285)	<0.001

ICU = intensive care unit; MGUS = monoclonal gammopathies of undetermined significance; NA = not applicable; pts = patients.

Variants of concern were available in 25 vaccinated patients: n = 2 Alfa (8%), n = 4 Delta (16%), and n = 19 Omicron BA.1 (76%). Despite the high vaccination coverage rate, most of the cases of positivity (n = 81; 94.2%) to SARS-CoV-2 were found between December, 2021, and January, 2022, mimicking what was observed in the general population.

About safety, from the start of vaccination campaign (December 27, 2020), no relevant or unexpected vaccine-related side effects were recorded in our court of 1086 MGUS vaccinated subjects until March 31, 2022.

Data regarding evaluation of humoral response after a complete course of anti-SARS-CoV-2 vaccine are yet limited in MGUS.^5–7,11^ In particular, we are not aware of previously published results specifically addressing the determination of anti-spike IgG antibodies with three doses of vaccine. Such an evaluation (after a median time of 100 days, range 45–180) was available in 20 of our MGUS, COVID-19-naive subjects. All these patients were judged as “responders,” as they achieved a titer greater than 50 AU (Arbitrary Units)/mL, which is considered the cutoff limit for response by the test manufacturer (Abbott). In particular, median value was 9050 AU/mL (range 1,482–54,390), a level that was quite similar to that observed in vaccinated age- and sex-matched HCs (data not shown).

To the best of our knowledge, this is the first report of “clinical” efficacy on COVID-19 of anti-SARS-CoV-2 vaccines in MGUS patients. Obviously, our study suffers from some limitations, such as the lack of information regarding protection provided by vaccines in the long term due to the short duration of follow-up, as well the scarcity of data regarding amount and durability of serological immune response. Notwithstanding, our observations highlight some relevant points. First, the incidence of SARS-CoV-2 infection was not significantly reduced in vaccinated MGUS patients, particularly in those who had received only 2 doses, probably because of a different pandemic scenario, characterized by higher diffusion capacity of the more recently recognized viral variants and fewer restriction measures applied during the last months. Three doses also significantly prolonged the time elapsed from vaccination to infection. Furthermore, the clinical outcome of COVID-19 appeared to be significantly improved by vaccines, particularly after 3 doses, thus supporting fully, extended vaccination programs also in patients with MGUS. Finally, we report here preliminary data about the apparently “normal” humoral response after 3 doses of anti-SARS-CoV-2 vaccines in MGUS. More patients and adequate follow-up will be necessary to evaluate the clinical significance of this finding, also in the light of the emerging issue of “hybrid” immunization (vaccines plus SARS-CoV-2 infection), recently reported in multiple myeloma.^12^

## AUTHOR CONTRIBUTIONS

PM and NS conceived and led the project. NS conducted database building, extraction and coding. NS and PM queried and analyzed the data. PM and NS wrote the main manuscript text and created all tables. All authors made a substantial intellectual contribution to the study, interpreted the data, discussed the results and reviewed, edited, and approved the final version of the manuscript.

## DISCLOSURES

The authors have no conflicts of interest to disclose.
